# Effects of exercise interventions on cancer-related fatigue in breast cancer patients: an overview of systematic reviews

**DOI:** 10.1007/s00520-022-07389-5

**Published:** 2022-11-03

**Authors:** Hong-Juan Zhou, Tao Wang, Yong-Zhi Xu, Yan-Nan Chen, Li-Jing Deng, Chang Wang, Jin-Xiu Chen, Jing-Yu (Benjamin) Tan

**Affiliations:** 1 School of Nursing, Putian University, 1133 Xueyuan Middle Road, Putian, Fujian China; 2 College of Nursing and Midwifery, Charles Darwin University, Brisbane Centre, 410 Ann Street, Brisbane, QLD Australia; 3 Department of Traditional Chinese Medicine, Putian Hospital of Traditional Chinese Medicine, 99 Xueyuan North Road, Putian, Fujian China; 4 School of Nursing, Fujian University of Traditional Chinese Medicine, 1 Qiu Yang Road, Fuzhou, Fujian China; 5 College of Nursing and Midwifery, Charles Darwin University, Ellengowan Dr, Casuarina, NT Australia

**Keywords:** Breast neoplasms, Exercise, Overview, Systematic reviews, Fatigue

## Abstract

**Objective:**

This overview of systematic reviews aims to critically appraise and consolidate evidence from current systematic reviews (SRs)/meta-analyses on the effects of exercise interventions on cancer-related fatigue (CRF) in breast cancer patients.

**Methods:**

SRs/meta-analyses that explored the effects of exercise interventions on CRF in breast cancer patients compared with the routine methods of treatment and care were retrieved from nine databases. The methodological quality of the included SRs was appraised using A MeaSurement Tool to Assess systematic Reviews II (AMSTAR II). The Grading of Recommendations Assessment, Development and Evaluation (GRADE) was used to calculate the grading of outcomes in the included SRs. The exercise type, frequency, duration, and inclusion/absence of supervision were further evaluated with subgroup analyses. The Stata 16.0 software was utilized for data analysis.

**Results:**

Twenty-nine reviews were included. The overall methodological quality and level of evidence of the included reviews were unsatisfactory, with only three reviews rated as high methodological quality and no review identified as high-quality evidence. Moderate certainty evidence indicated that exercise could improve fatigue in breast cancer patients (*SMD* = − 0.40 [95%CI − 0.58, − 0.22]; *P* = 0.0001). Subgroup analysis based on the types of exercise showed that yoga (*SMD* = − 0.30 [95%CI − 0.56, − 0.05]; *I*^2^ = 28.7%) and aerobic exercise (*SMD* = − 0.29 [95%CI − 0.56, − 0.02]; *I*^2^ = 16%) had a significantly better effect on CRF in breast cancer patients; exercising for over 6 months (*SMD* = − 0.88 [95%CI − 1.59, − 0.17]; *I*^2^ = 42.7%; *P* = 0.0001), three times per week (*SMD* = − 0.77 [95%CI − 1.04, − 0.05]; *I*^2^ = 0%; *P* = 0.0001), and for 30 to 60 min per session (*SMD* = − 0.81 [95%CI − 1.15, − 0.47]; *I*^2^ = 42.3%; *P* = 0.0001) can contribute to a moderate improvement of CRF. Supervised exercise (*SMD* = − 0.48 [95%CI − 0.77, − 0.18]; *I*^2^ = 87%; *P* = 0.001) was shown to relieve CRF.

**Conclusion:**

Exercise played a favorable role in alleviating CRF in breast cancer. Yoga was recommended as a promising exercise modality for CRF management in the majority of the included studies. Exercising for at least three times per week with 30 to 60 min per session could be recommended as a suitable dosage for achieving improvement in CRF. Supervised exercise was found to be more effective in alleviating CRF than unsupervised exercise. More rigorously designed clinical studies are needed to specify the exact exercise type, duration, frequency, and intensity to have an optimal effect on CRF in breast cancer patients.

**Trial registration:**

ClinicalTrials.gov Identifier: CRD42020219866.

**Supplementary Information:**

The online version contains supplementary material available at 10.1007/s00520-022-07389-5.

## Introduction

The Global Burden of Disease Study 2020 [1] indicated that breast cancer remains the leading cancer diagnosis among females [2]. Although survival rates of breast cancer are improving, patients still experience a series of adverse effects caused by cancer and its related treatment, such as depression, fatigue, sleep disturbance, and bone marrow suppression [3, 4]. Cancer-related fatigue (CRF) is one of the most familiar and often overlooked symptoms [5], referring to a general, persistent, and subjective feeling of fatigue caused by cancer or relevant treatment that cannot be improved by sleep or rest [6] and may persist for months or even years [7]. The incidence of CRF in breast cancer patients is higher than that in other types of cancers [1], among which up to 33% of patients experience fatigue five years after the end of breast cancer treatment [8]. CRF adversely affects breast cancer patients in multiple aspects [9], which severely not only affects their quality of sleep but also prolongs their length of hospital stay and can result in a reduction of physical, mental, and emotional function and poor quality of life [10].

Currently, some pharmaceutical agents such as stimulants, antidepressants, acetylcholinesterase inhibitors, and corticosteroids have been recommended for CRF management in breast cancer patients [10] However, those pharmacological approaches were reported to be associated with a range of undesirable side effects such as tumor protection [11], decreased appetite [12] and venthrombotic events [13, 14]. The unclear pathophysiological mechanism of CRF also makes it difficult to develop tailored pharmacological interventions for CRF management [6]. Non-pharmacological interventions such as exercise interventions [10], mindfulness-based decompression therapy [15], and cognitive behavioral therapy [16] have been explored as adjuvant approaches to pharmacological interventions to alleviate CRF. Exercise interventions refer to a physical activity treatment that is planned, structured, and repetitive and have a final or an intermediate objective of improving or maintaining physical fitness, which includes running, aerobics, tai chi, yoga, and resistance exercise [17]. Exercise interventions have been commonly utilized and recommended as an effective intervention for the alleviation of CRF by the American Society of Clinical Oncology (ASCO) [18] and Exercise and Sports Science Australia (ESSA) [19]. In addition, the Japan Breast Cancer Society (JBCS) [20], the German Gynecological Oncology Group (AGO) [21], and a previous systematic review [22] found that exercise interventions are an effective, low-risk modality for breast cancer patients in reducing morbidity and improving body functions and quality of life. However, most of the literature on exercise interventions [18, 19, 21] have not clearly stated the type, frequency, and duration of exercise for practice in breast cancer patients with CRF, leading to a gap in developing personalized and evidence-based exercise intervention protocols tailored to patients’ health conditions and needs.

With the rapid development of evidence-based medicine in the field of cancer supportive care, an increasing body of systematic reviews (SRs)/meta-analyses have provided much evidence on using exercise interventions for CRF management in breast cancer patients, but their conclusions were inconsistent [23, 24] and the methodological quality varied across studies, which are barriers to the transformation of research evidence to practice and the application of clinical decision-making. To our knowledge, no overviews of systematic reviews on the effects of exercise interventions on CRF in breast cancer patients have been conducted so far. Thus, the aim of this overview was to critically appraise and consolidate evidence from current SRs/meta-analyses on the effects of exercise interventions on CRF in breast cancer patients. Specifically, the study objectives were as follows: (1) to identify the effects of exercise interventions on relieving CRF in breast cancer patients; (2) to assess the methodological quality of as well as the level of evidence from current SRs/meta-analyses on the effects of exercise interventions for breast cancer patients with CRF; and (3) to identify the optimal modality, duration, and frequency of exercise interventions for CRF management in breast cancer patients.

## Methods

This overview of systematic reviews was reported in accordance with the Preferred Reporting Items for OoSRs (PRIO-harms) checklist and the Preferred Reporting Items for OoSRs (PRIO) checklist. The protocol has been registered with PROSPERO (CRD42020219866). A pre-print version of this manuscript is also available at https://www.researchsquare.com/article/rs-1376171/v1

### Data sources and searches

This overview included SRs/meta-analyses that focused on the effects of exercise therapy on CRF in breast cancer patients. Relevant SRs/meta-analyses were comprehensively searched until September 2021 through the following data sources: (1) PubMed, Cochrane Library, Excerpta Medica Database (EMBASE), Web of Science, China National Knowledge Infrastructure (CNKI), China Biology Medicine Disc (CBMdisc), Wan Fang Data, and China Science and Technology Journal Database, and The Lancet; (2) references of the included SRs/meta-analyses; and (3) grey literature from the National Institute for Health Research (NIHR) Centre, such as unpublished manuscripts and published reports. The search terms included “breast neoplasms”, “exercise therapies”, “fatigue”, “systematic review”, and “meta-analysis”. The search procedure in the databases above followed the text string “((Breast neoplasms) OR (Breast tumor) OR (Mammary cancer) OR (Breast cancer) OR (Carcinoma breast)) AND (Exercise OR (Physical activity) OR (Physical exercise) OR (Exercise training) OR (Exercise therapies)) AND (Fatigue OR CRF) AND ((Systematic review) OR meta-analysis)”. Taking PUBMED and EMBASE as examples, a full search strategy was summarized in Supplementary file A.

### Inclusion and exclusion criteria

The inclusion criteria were developed in accordance with the Population, Intervention, Comparator, Outcome, Study (PICOS) framework: (1) types of studies: SRs/meta-analyses of randomized controlled trials (RCTs) that were published in either English or Chinese; (2) types of populations: adult breast cancer patients [5] with CRF [25], regardless of stages of cancer, age, gender, and nationality; (3) types of interventions: exercise interventions [17], such as aerobic exercise, tai chi, yoga, resistance training, dancing, and walking; (4) types of comparison: routine methods of treatment and care with no active exercise components or any other types of active treatments; and (5) types of outcomes: CRF as the primary outcome as measured by valid assessment tools, such as the Functional Assessment of Chronic Illness Therapy-Fatigue (FACIT-F) Scale, the Multidimensional Fatigue Inventory (MFI), or the Brief Fatigue Inventory (BFI). Exclusion criteria were the following: (1) proposals of SRs or meta-analyses; (2) study population was breast cancer mixed with other diseases or complications; (3) conference abstracts; (4) full text was not available after multiple search methods, including contacting the author.

### Literature screening and data extraction

**Fig. 1:**
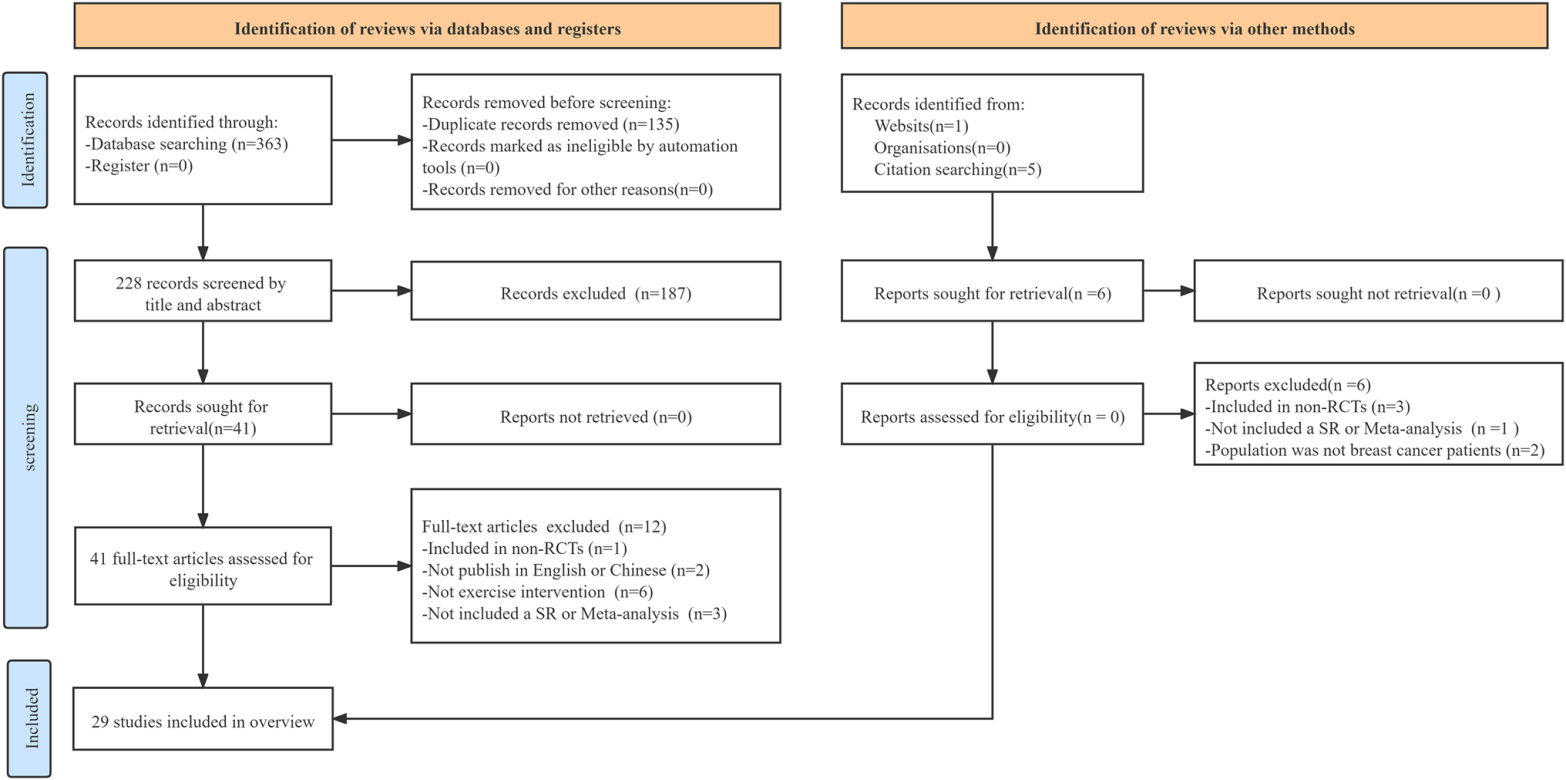
Flowchart of the study selection process (PRISMA diagram)

Duplications were identified and removed via reference management software (NoteExpress). The titles and abstracts of the rest of the SRs/meta-analyses were screened by two reviewers (HJZ and YZX) independently to determine the potentially eligible SRs/meta-analyses. Full texts of the potentially eligible SRs/meta-analyses were further screened and examined by the same two reviewers. If there were duplications, the latest version of the SR or meta-analysis was selected. Eligible SRs/meta-analyses were finally included after discussion between the reviewers. Any contradiction regarding study inclusion was resolved through consultation or arbitration by an experienced third reviewer (TW). Data from the included SRs/meta-analyses were extracted using a data extraction form predesigned by one reviewer (HJZ), which was verified by another reviewer (YZX). Disagreements between the two reviewers regarding data extraction were discussed by involving a third reviewer (TW). The extracted data included the author, publication year and country, number of studies and sample size of the participants, types of intervention and control, quality assessment (whether the included SRs/ meta-analyses evaluated the quality of their included studies and the tools used for the quality appraisal), measurement tools, main conclusion, and whether it included a meta-analysis. Moreover, relevant data for subgroup analysis including the exercise type, frequency, duration, and inclusion/absence of supervision were extracted and verified. The study selection process is presented in Fig. 1.

### Quality appraisal of the included reviews

The methodological quality and the level of evidence of the included SRs/meta-analyses were independently assessed by two reviewers (HJZ and YZX) with two tools (see the “Methodological quality” and the “Evidence quality” sections). The final assessment results were cross-checked. Any disapprovals were discussed and decided by involving a third reviewer (JYT).

#### Methodological quality

A Measurement Tool to Assess systematic Reviews II (AMSTAR II) was used to comprehensively assess the methodological quality of the included SRs/meta-analyses [26], which is presently the most widely used methodological quality assessment tool [27, 28]. The AMSTAR II includes 16 items (www.amstar.ca), each of which can be answered “yes” or “no”, and some of the items can be answered “partially yes” [27, 28]. Seven items, including items 2, 4, 7, 9, 11, 13, and 15, that are considered to critically affect the validity of the included reviews and its conclusions are generally recommended as critical items [26]. The methodological quality of the included reviews was rated using the following criteria: (1) high quality: no or only one non-critical item flaw; (2) moderate quality: more than one non-critical item flaw but no critical item flaws; (3) low quality: one critical item flaw, with or without a non-critical item flaw; and (4) critically low quality: more than one critical item flaw, with or without a non-critical item flaw [26, 27, 29].

#### Evidence quality

Two reviewers (HJZ and YZX) used the Grading of Recommendations Assessment, Development and Evaluation (GRADE) to rate the level of evidence of the included SRs/meta-analyses in five aspects, including limitations, inconsistencies, indirectness, imprecision, and publication bias [30]. Disagreements were addressed by involving a third author (TW) until consensus was achieved. For each aspect, the evidence was graded as high, moderate, low, or extremely low. Detailed grading criteria were as follows [31]: (1) high-level evidence: not downgraded, which represents the true effect estimates; (2) moderate-level evidence: downgraded one grade, which indicates that the true value is possible to come near to the estimate but is substantially different; (3) low-level evidence: downgraded two grades, which indicates that there is a significant difference between the actual and estimated values; and (4) extremely low-level evidence: downgraded three grades, which indicates that the true value is likely to be very different from the estimated value.

### Data analysis

The characteristics of the included SRs, including author, publication year and country, number of studies and sample size, types of intervention and control, and main findings (i.e., effects of the exercise on CRF), are summarized in Table 1. The overlap across the included studies (only RCTs) of the analyzed SRs/meta-analyses was estimated using the corrected covered area (CCA) [32]. A lower CCA value indicated a lower likelihood of overlaps [32]. A CCA value of 5% or below was regarded as a “slight overlap”, 6–10% as a “moderate overlap”, 11–15% as a “high overlap”, while above 15% was regarded as a “very high overlap” [33]. For continuous variables, mean differences (MD) or standardized mean differences (*SMD*) with 95% confidence intervals (CI) was used for meta-analysis and effect size calculation (a *P* value of ≤ 0.05 was considered statistically significant). For continuous variables, the random-effects model was used to calculate the number of participants and RCTs included in the meta-analyses and to summarize the effect size [with 95% confidence intervals (CI) and *P* values ≤ 0.05 considered significant]. According to Cohen [33], 0.2 is considered a small effect, 0.2 to 0.8 a medium effect, and 0.8 or above a large effect. We also extracted and analyzed the data of included meta-analyses to better illustrate the effects of the duration of the interventions, exercise type, frequency, and duration of each session on CRF of breast cancer patients. Because of the lack of relevant data, direct comparisons between different interventions were impossible. *I*-square (*I*^2^) statistics were used to measure the heterogeneity of the included SRs/meta-analyses and explain the various thresholds by effect size and direction and the *P*-value from Cochran’s Q test [34]. An *I*^2^ value > 50% is regarded as a substantial level of heterogeneity [34]. Sub-group analyses are planned based on exercise type, frequency, and duration, and inclusion/absence of supervision. Statistical analysis was conducted with the Stata version 16.0 software.

**Table 1:** Characteristics of the 29 SRs/meta-analyses

First author	Year	Country	Number of studies (partici-pants)	Interventions		Quality assessment	Measurement tools	Main conclusion	Meta-analysis
				Treatment	Control				
Ehlers [36]	2020	USA	22 (2348)	Aerobic exercise Resistance exercise Yoga Qi gong	Routine methods of care No exercise	RoB	FACIT-F; BFI; MFI; FAQ; PFS	Exercise intervention improved CRF in breast cancer patients after chemotherapy, but the effect was small to moderate	Yes
Shen [37]	2020	PR China	8 (782)	Exercise	Routine methods of care Relaxation control	RoB	FACIT-F; BFI; FAQ	CRF, pain, and depression were significantly reduced by exercise interventions for breast cancer patients undergoing radiotherapy	Yes
Lee [38]	2018	Korea	8 (826)	Exercise	Routine methods of care No exercise	Quality assessment of controlled intervention studies	MFI; RPFS	Aerobic exercise, resistance training, and a combination of exercises produced beneficial effects on CRF, and the effect of high-intensity exercise was better than that of low- or moderate-intensity exercise	Yes
Singh [39]	2018	Australia	31 (2878)	Aerobic exercise Resistance exercise Strength exercise Pilates	Routine methods of care	RoB	FACIT-F; PFS; SCFS-6; POMS-F; BFI; MFI; FAQ	Most of the breast cancer patients safely participated in the exercise interventions at moderate- or high-intensity, which improved their CRF	Yes
Lipsett [40]	2017	Ireland	9 (755)	Resistance exercise Strength exercise Yoga	Routine methods of care	PEDro	FAQ; FACIT-F; PFS; BFI	Exercise may be beneficial for CRF management in breast cancer patients undergoing radiotherapy. Supervised exercise intervention was more effective in alleviating CRF than unsupervised exercise	Yes
Juvet [41]	2017	Norway	17 (1812)	Aerobic exercise Resistance exercise	Routine methods of care	NOKC	FACIT-F; PFS; SCFS-6; POMS-F; MFI; PFI	Breast cancer patients’ CRF was reduced by regular exercise, which had a positive effect, but the effect was not significant	Yes
Zhu [23]	2016	PR China	12 (1574)	Aerobic exercise Resistance exercise Yoga	Routine methods of care No exercise Supportive therapy	RoB	FACIT-F; MFI; VO_2_ Peak	The effect of exercise on the improvement of CRF in breast cancer patients was not significant	Yes
McNeely [42]	2006	Canada	5 (230)	Exercise	Routine methods of care	Homemade Standard	FACIT-F; PFS	Exercise improved CRF in breast cancer patients	Yes
Gu [43]	2012	PR China	9 (988)	Aerobic exercise Gymnastics	Routine methods of care	RoB	FACIT-F; FS; POMS-F; LAS-F	There was a significant difference of CRF in breast cancer patients between the exercise intervention groups and the control groups	Yes
Vannorsdall [24]	2020	USA	11 (1654)	Exercise	Routine methods of care	RoB	PFS; CFS; MFI;FAQ;	The exercise interventions had a remarkable effect on CRF in breast cancer patients during and after treatment	Yes
van Vulpen [44]	2016	The Nether-lands	5 (707)	Exercise	Routine methods of care	RoB	FAQ; MFI	Physical exercise had the greatest effect on reducing body fatigue, as the importance of auxiliary exercise was emphasized	Yes
Ramírez-Vélez [45]	2021	Republic of Lithuania	39 (4150)	Aerobic exercise Resistance exercise	Routine methods of care	PEDro	FACIT-F; BFI; MFI	Exercise had a moderate positive effect on CRF in breast cancer patients. Each session of tai chi lasted > 40 min, and the intervention duration was ≤ 6 weeks, which had a good relief effect on CRF	Yes
Liu [46]	2021	PR China	17 (1133)	Tai chi Yoga Strength exercise Pilates	Routine methods of care Health education	RoB	BFI; EORTC-QLQ-C30; FACIT-F; CFS; FSI; PFS-R	Mind–body exercise improved CRF in breast cancer patients	Yes
Lin [47]	2021	Taiwan, China	9 (581)	Aerobic Yoga	Routine methods of care	JBI-MAStARI	PFS; FSI; PFS-R; FACIT-F; SCFS	Breast cancer patients benefitted from exercise, including reduced fatigue. In addition, supervised exercise by a nurse or family member had a stronger effect on CRF in breast cancer patients	Yes
Duijts [48]	2011	The Nether-lands	11 (1000)	Resistance exercise Yoga	Routine methods of care Health education	None	FACIT-F; MFI; FS; LAS-F; PFS; POMS-F; FSS	CRF was reduced by physical exercise, but the overall effect warrants further investigation	Yes
Liu [49]	2017	PR China	4 (674)	Exercise	Routine methods of care Walking	None	FACIT-F	CRF in breast cancer patients was reduced by home-based physical exercise, which should be recommended	Yes
Zheng [50]	2021	PR China	5 (436)	Yoga	Routine methods of care	RoB	CFS	Fatigue in breast cancer patients was reduced by exercise, such as yoga and qi gong	Yes
Wu [51]	2018	PR China	3 (400)	Yoga	Routine methods of care	RoB	FACIT-F; CFS	For breast cancer patients, CRF was reduced by yoga, but more large, high-quality studies are needed	Yes
Zhang [52]	2015	PR China	9 (623)	Yoga	Routine methods of care Health education No exercise	RoB	FACIT-F; FSI	Yoga was positively significant in alleviating CRF in patients with breast cancer	Yes
O’Neill [53]	2020	Canada	24 (1379)	Yoga	Routine methods of care Health education No exercise	RoB	FACIT-F; FSI; BFI; EORTC-QLQ-C30	Based on the available evidence, yoga reduced CRF in breast cancer patients, with effects comparable to those of aerobic exercise	Yes
Dong [54]	2019	PR China	17 (2273)	Yoga	Routine methods of care	RoB	BFI; FSS; CFS; EORTC-QLQ-C30; FACIT-F; MFI; MFSI-SF	The breast cancer patients’ physical fatigue was alleviated by yoga, which had a moderate effect on cognitive fatigue and a relatively small effect on mental fatigue	Yes
Hsueh [55]	2021	Taiwan, China	14 (882)	Yoga	Routine methods of care Health education	RoB 2.0	BFI; FACIT-F; MFI; MFSI-SF	CRF in breast cancer patients can be lessened by yoga without serious adverse events, and yoga was strongly recommended as a supportive therapy for CRF post-treatment	Yes
Xu [56]	2020	PR China	15 (1650)	Aerobic exercise (walking, dancing, swimming, etc.)	Routine methods of care	RoB	RPFS; PFS; POMS-F; FACIT-F	Aerobic exercise had an effect on CRF in breast cancer patients, but the mechanism was not fully understood	Yes
Hu [57]	2009	PR China	6 (494)	Aerobic	Routine methods of care No exercise	RoB	FACIT-F; PFS	Rehabilitation exercise was initially shown to be effective in alleviating CRF in breast cancer patients undergoing chemotherapy/radiotherapy	Yes
Yang [58]	2019	PR China	12 (1232)	Aerobic exercise (jogging, cycling, swimming, etc.)	Routine methods of care No exercise	RoB	PFS; BFI; MFI; FSS; FSI; FACIT-F	CRF was relieved by aerobic exercise. However, aerobic exercise was difficult to achieve in the double-blind clinical intervention, and thus the Hawthorne effect may have occurred, affecting the authenticity of the results	Yes
Zhang [59]	2012	PR China	9 (593)	Aerobic exercise	Routine methods of care	Jadad Scores	PFS; SCFS-6; FACIT-F; MFI	Aerobic exercise had an influence on CRF, with degree related to the duration of the exercise intervention	Yes
Zou [60]	2014	PR China	12 (1104)	Aerobic exercise	Routine methods of care	NOS	FACIT-F; PFS	Aerobic exercise effectively improved CRF caused by chemotherapy in breast cancer patients	Yes
Luo [61]	2020	PR China	3 (160)	Tai chi	Routine methods of care Supportive therapy	RoB	FACIT-F	Tai chi quan exercise had positive effects on CRF remission, but more rigorous approaches and low risk-of-bias RCTs should be provided to support more reliable evidence	Yes
Liu [62]	2019	New Zealand	2 (337)	Tai chi	Routine methods of care	PEDro	FACIT-F; FSI	Due to the small number of included studies, it was impossible to verify the effect of tai chi in breast cancer patients with CRF	Yes

*RoB*, Cochrane risk-of- bias criteria; *NOKC*, Norwegian Knowledge Centre for the Health Services Handbook for Systematic Reviews; *PEDro*, Physiotherapy Evidence Databases Scale; *NOS*, Newcastle–Ottawa Scale; *JBI-MAStARI*, Joanna Briggs Institute-Critical Appraisal Tool for Randomized Controlled Trials; *RoB 2.0*, Revised Risk-of-Bias Tool for Randomized Trials; *FAQ*, Fatigue Assessment Questionnaire; *RPFS*, Revised Piper Fatigue Scale; *PFI*, Peripheral Fatigue Instrument; *SCFS-6*, Schwartz Cancer Fatigue Scale-6; *POMS*-*F*, Profile of Mood States Fatigue Scale; *FS*, Fatigue Scale; *LAS-F*, Linear Analog Scale for Fatigue; *CFS*, Cancer Fatigue Scale; *FSI*, Fatigue Symptom Inventory; *PFS-R*, the Revised Piper Fatigue Scale; *EORTC-QLQ-C30*, Eastern Co-operative Oncology Group Quality of Life Questionnaire-C30 Fatigue Subscale; *FSS*, Fatigue Severity Scale; *MFSI-SF*, Multidimensional Fatigue Symptom Inventory-Short Form; *VO*_*2*_* Peak*, Peak Oxygen Uptake

Adapted from: Preferred reporting items for overviews of systematic reviews [35].

## Results

### Identification of the included reviews

A total of 369 records were searched, of which 135 were excluded due to duplication and 193 were screened by title or abstract and deemed irrelevant to the topic. Of the remaining 41 records, 12 were excluded after assessing the full text for eligibility. Twenty-nine SRs/meta-analyses [23, 24, 36–62] were finally included in the overview. The literature retrieval and selection process are shown in Fig. 1. A list of excluded reviews from full-text analysis with reasons is provided in supplementary file B.

### Characteristics of the included reviews

The 29 SRs/meta-analyses included 402 studies, with a total of 33,655 patients, published between 2006 and 2021. Meta-analyses were carried out for all the included reviews. A total of 252 RCTs were included and analyzed across the 29 reviews, with a CCA of 3% indicating a slight overlap rate that reflected a low level of unnecessary duplications in the reviews and less biased results. Sixteen reviews [23, 24, 36–49] explored the effects of exercise therapy on CRF by including studies with different types of exercise, including aerobic exercise, yoga, resistance exercise, and Pilates. For the other 13 reviews, six [50–55] focused on yoga, five [56–60] on aerobic exercise, and two [61, 62] on tai chi. Routine methods of care and/or health education without any active exercise components were commonly utilized as the study comparisons. Seventeen reviews [23, 24, 36, 37, 39, 43, 44, 46, 50–54, 56–58, 61] used the Cochrane risk of bias (RoB) criteria. Other reviews were assessed using the Quality Assessment of Controlled Intervention Studies [38], the Norwegian Knowledge Centre for the Health Services (NOKC) Handbook for Systematic Reviews [41], Homemade Standard [42], the Revised Risk-of-Bias Tool for Randomized Trials (RoB 2.0) [55], Jadad Scores [59], the Newcastle–Ottawa Scale (NOS) [60], the Physiotherapy Evidence Databases (PEDro) Scale [40, 45, 62], and the Joanna Briggs Institute-Critical Appraisal for Randomized Controlled Trials (JBI-MAStARI) tool [47], respectively. Two reviews [48, 49] did not describe its methodological quality assessment process. The FACIT-F Scale [23, 36, 37, 39–43, 45–49, 51–62], the BFI [36, 37, 39, 40, 45, 46, 53–55, 58], the MFI [23, 24, 36, 38, 39, 41, 44, 45, 48, 54, 55, 58, 59], and the Piper Fatigue Scale (PFS) [24, 36, 39–42, 47, 48, 56–60] were the most commonly used instruments for CRF assessment in the 29 SRs/meta-analyses, the characteristics of which are presented in Table 1.

### Quality appraisal of included reviews

#### Methodological quality

Regarding the methodological quality of the included SRs/meta-analyses, three reviews [40, 45, 53] were evaluated as high quality, 21 reviews [23, 24, 36, 37, 39, 41, 43, 44, 46–48, 50–52, 54, 56–58, 60–62] were rated as low quality, and the remaining five reviews [38, 42, 49, 55, 59] were assessed as critically low quality. Specifically, the critical items that had an effect on the quality of the reviews were item 2 (only five reviews [45, 46, 50, 55, 61] were evaluated as “yes” due to registered proposals in the early stage, and the remaining reviews only provided the research methods so they were assessed as “partly yes”, which means that the research methods could not be compared with the registered proposals approved by official organizations and may have caused a risk of bias), item 4 (whether to search for grey literature and counsel experts in the relevant field was not mentioned in 18 reviews[23, 24, 36, 37, 39, 43–45, 48–51, 54–57, 59, 60], suggesting that there may have been incomplete retrievals in the above, which may have led to results and conclusion errors), and item 7 (apart from three reviews[40, 45, 53], the list of excluded references and the causes for their exclusion were not provided and illustrated in the other reviews, which reduced the rigor of the study and the reliability of the results). In addition, non-critical item 10 also affected the methodological quality results since none of the 29 reviews reported the funding of their included RCTs, which indicated uncertainty about the possibility of commercial funding interference that might have made study results favorable to the commercial funder. All the reviews described the basic characteristics of and were able to scientifically discuss and analyze the included studies. Specific methodological quality assessment results are shown in Table 2.

**Table 2:** Methodological quality of the 29 SRs/meta-analyses

Reviews	AMSTAR II																Overall quality
	Q1	Q2	Q3	Q4	Q5	Q6	Q7	Q8	Q9	Q10	Q11	Q12	Q13	Q14	Q15	Q16	
Ehlers [36]	Y	P	Y	Y	Y	Y	N	Y	Y	N	Y	Y	Y	Y	Y	Y	Low
Shen [37]	Y	P	Y	P	Y	Y	N	Y	Y	N	Y	Y	Y	Y	Y	Y	Low
Lee [38]	Y	P	Y	N	Y	Y	N	Y	N	N	Y	N	N	Y	N	Y	Critically low
Singh [39]	Y	P	Y	P	N	Y	N	Y	Y	N	Y	Y	Y	Y	Y	Y	Low
Lipsett [40]	Y	P	Y	Y	Y	Y	Y	Y	Y	N	Y	Y	Y	Y	Y	Y	High
Juvet [41]	Y	P	Y	Y	Y	Y	N	Y	Y	N	Y	Y	Y	Y	Y	Y	Low
Zhu [23]	Y	P	Y	P	Y	Y	N	Y	Y	N	Y	Y	Y	Y	Y	Y	Low
McNeely [42]	Y	P	Y	Y	Y	Y	N	Y	Y	N	Y	N	N	Y	Y	Y	Critically low
Gu [43]	Y	P	Y	P	N	N	N	Y	Y	N	Y	Y	Y	Y	Y	Y	Low
Vannorsdall [24]	Y	P	Y	P	Y	Y	N	Y	Y	N	Y	Y	Y	Y	Y	Y	Low
van Vulpen [44]	Y	P	Y	P	Y	Y	N	Y	Y	N	Y	Y	Y	Y	Y	Y	Low
Ramírez-Vélez [45]	Y	Y	Y	P	Y	Y	Y	Y	Y	N	Y	Y	Y	Y	Y	Y	High
Liu [46]	Y	Y	Y	Y	Y	Y	N	Y	Y	N	Y	Y	Y	Y	Y	Y	Low
Lin [47]	Y	P	Y	Y	Y	Y	N	Y	Y	N	Y	Y	Y	Y	Y	Y	Low
Duijts [48]	Y	P	Y	P	Y	Y	N	Y	Y	N	Y	Y	Y	Y	Y	Y	Low
Liu [49]	Y	P	N	P	Y	Y	N	Y	N	N	Y	Y	Y	Y	Y	Y	Critically low
Zheng [50]	Y	Y	Y	P	Y	Y	N	Y	Y	N	Y	Y	Y	Y	Y	N	Low
Wu [51]	Y	P	Y	P	Y	N	N	Y	Y	N	Y	Y	Y	Y	Y	N	Low
Zhang [52]	Y	P	Y	Y	Y	Y	N	Y	Y	N	Y	Y	Y	Y	Y	N	Low
O’Neill [53]	Y	P	Y	Y	Y	Y	Y	Y	Y	N	Y	Y	Y	Y	Y	Y	High
Dong [54]	Y	P	Y	P	Y	Y	N	Y	Y	N	Y	Y	Y	Y	Y	Y	Low
Hsueh [55]	Y	Y	Y	Y	Y	Y	N	Y	Y	N	Y	N	Y	Y	N	Y	Critically low
Xu [56]	Y	P	Y	P	Y	Y	N	Y	Y	N	Y	Y	Y	Y	Y	Y	Low
Hu [57]	Y	P	Y	P	Y	N	N	Y	Y	N	Y	Y	Y	Y	Y	Y	Low
Yang [58]	Y	P	Y	Y	Y	Y	N	Y	Y	N	Y	Y	Y	Y	Y	N	Low
Zhang [59]	Y	P	Y	P	Y	Y	N	Y	N	N	Y	Y	Y	N	N	N	Critically low
Zou [60]	Y	P	Y	P	Y	Y	N	Y	Y	N	Y	Y	Y	Y	Y	Y	Low
Luo [61]	Y	Y	Y	P	Y	Y	N	Y	Y	N	Y	Y	Y	Y	Y	Y	Low
Liu [62]	Y	P	Y	Y	Y	Y	N	Y	Y	N	Y	Y	Y	Y	Y	Y	Low
P + Y	100%	100%	96.6%	96.6%	93.1%	89.7%	10.3%	100%	89.7%	0.0%	100%	89.7%	93.1%	96.6%	89.7%	82.8%	

Q1. “Did the research questions and inclusion criteria for the review include the components of PICO?”; Q2. “Did the report of the review contain an explicit statement that the review methods were established prior to the conduct of the review and did the report justify any significant deviations from the protocol?”; Q3. “Did the review authors explain their selection of the study designs for inclusion in the review?”; Q4. “Did the review authors use a comprehensive literature search strategy?; Q5. “Did the review authors perform study selection in duplicate?”; Q6. “Did the review authors perform data extraction in duplicate?”; Q7. “Did the review authors provide a list of excluded studies and justify the exclusions?”; Q8. “Did the review authors describe the included studies in adequate detail?”; Q9. “Did the review authors use a satisfactory technique for assessing the risk of bias (RoB) in individual studies that were included in the review?”; Q10. “Did the review authors report on the sources of funding for the studies included in the review?”; Q11. “If meta-analysis was performed did the review authors use appropriate methods for statistical combination of results?”; Q12. “If meta-analysis was performed, did the review authors assess the potential impact of RoB in individual studies on the results of the meta-analysis or other evidence synthesis?”; Q13. “Did the review authors account for RoB in individual studies when interpreting/ discussing the results of the review?”; Q14. “Did the review authors provide a satisfactory explanation for, and discussion of, any heterogeneity observed in the results of the review?”; Q15. “If they performed quantitative synthesis did the review authors carry out an adequate investigation of publication bias (small study bias) and discuss its likely impact on the results of the review?”; Q16. “Did the review authors report any potential sources of conflict of interest, including any funding they received for conducting the review?”(Shea BJ,2017 [26] p.3–5)

Answers: *Y*, yes; *P*, partial yes; *N*, no

#### Evidence quality

Eleven reviews [37, 38, 40, 43, 49–52, 59, 60, 62] were evaluated as having an extremely low level of evidence, 13 reviews [23, 24, 42, 44, 46–48, 53, 54, 56–58, 61] had a low level of evidence, and the remaining five [36, 38, 39, 41, 45] had a moderate level of evidence. Inconsistency (*n* = 22, 68.75%) was the most common reason for downgrading levels in the included reviews, followed by publication bias (*n* = 17, 53.12%), limitations (*n* = 15, 46.8%), imprecision (*n* = 10, 31.25%). Elaborating on the reasons for downgrading levels, the most common reason was the significant heterogeneity of the results [23, 36, 37, 39–43, 45–47, 49–57, 59, 61, 62] (*n* = 23) and the missing grey literature and manual retrieval [24, 37, 40, 42, 44, 50, 57–59, 61, 62] (*n* = 11). Other reasons included an unclear description of the blinding procedures [36, 40, 50, 52, 53, 55, 61, 62] (*n* = 8), inclusion of invalid values (RR = 1.0) within the confidence intervals [23, 42, 49, 51, 52, 59, 60, 62] (*n* = 8), failure to report publication bias [38, 42, 48, 49, 51, 62] (*n* = 6), unsatisfactory methodological quality of the included RCTs [24, 43, 44, 52, 53] (*n* = 5), unreported or incomplete report of outcomes such as adverse reactions [58] (*n* = 1), and the inclusion of only one RCT resulting in an inability to measure heterogeneity [42] (*n* = 1). The results of the evidence assessment are presented in Table 3.

**Table 3:** Evidence quality of the 29 SRs/meta-analyses

Reviews	GRADE					Quality
	Limitation	Inconsistency	Indirectness	Imprecision	Publication bias	
Ehlers [36]	− 1^1^	0	0	0	0	⊕⊕⊕Ο Moderate
Shen [37]	0	− 1^2^	0	− 1^3^	− 1^4^	⊕ΟΟΟ Very low
Lee [38]	0	0	0	0	− 1^5^	⊕⊕⊕Ο Moderate
Singh [39]	0	− 1^2^	0	0	0	⊕⊕⊕Ο Moderate
Lipsett [40]	− 1^1^	− 1^2^	0	0	− 1^4^	⊕ΟΟΟ Very low
Juvet [41]	0	− 1^2^	0	0	0	⊕⊕⊕Ο Moderate
Zhu [23]	0	− 1^2^	0	− 1^3, 6^	0	⊕⊕ΟΟ Low
McNeely [42]	0	− 1^2, 6^	0	0	− 1^4^	⊕⊕ΟΟ Low
Gu [43]	− 1^1, 8^	− 1^2^	0	− 1^3^	0	⊕ΟΟΟ Very low
Vannorsdall [24]	− 1^8^	0	0	0	− 1^4^	⊕⊕ΟΟ Low
van Vulpen [44]	− 1^8^	0	0	0	− 1^4^	⊕⊕ΟΟ Low
Ramírez-Vélez [45]	0	− 1^2^	0	0	0	⊕⊕⊕Ο Moderate
Liu [46]	0	− 2^2^	0	0	0	⊕⊕ΟΟ Low
Lin [47]	0	− 2^2^	0	0	0	⊕⊕ΟΟ Low
Duijts [48]	0	0	0	0	− 1^5^	⊕⊕ΟΟ Low
Liu [49]	0	− 2^2^	0	− 1^6^	− 1^4, 5^	⊕ΟΟΟ Very low
Zheng [50]	− 1^1^	− 1^2^	0	0	− 1^4^	⊕ΟΟΟ Very low
Wu [51]	0	− 1^2^	0	− 1^6^	− 1^5^	⊕ΟΟΟ Very low
Zhang [52]	− 1^1, 8^	− 2^2^	0	− 1^6^	0	⊕ΟΟΟ Very low
O’Neill [53]	− 1^1^	− 1^8^	0	0	0	⊕⊕ΟΟ Low
Dong [54]	0	− 1^2^	0	0	0	⊕⊕ΟΟ Low
Hsueh [55]	− 1^1^	− 2^2^	0	0	0	⊕ΟΟΟ Very low
Xu [56]	0	− 1^2^	0	0	0	⊕⊕ΟΟ Low
Hu [57]	0	− 1^2^	0	0	− 1^4^	⊕⊕ΟΟ Low
Yang [58]	− 1^7^	0	0	0	− 1^4^	⊕⊕ΟΟ Low
Zhang [59]	0	− 1^2^	0	− 1^6^	− 1^4^	⊕ΟΟΟ Very low
Zou [60]	− 1^2^	− 2^3^	0	− 1^6^	0	⊕ΟΟΟ Very low
Luo[61]	− 1^1^	0	0	0	− 1^4^	⊕⊕ΟΟ Low
Liu[62]	− 1^1^	0	0	− 1^6^	− 1^5^	⊕ΟΟΟ Very low

^1^The included reviews were biased in terms of randomization, allocation concealment, and blinding method; ^2^the confidence intervals of different studies overlapped greatly, and the combined result of heterogeneity was large (> 80%, decreased by two grades); ^3^significant benefits or harms were included in the confidence interval (RR < 0.75 or *RR* > 1.25 were the criteria); ^4^whether gray literature and manual retrieval were included was not stated in the review; ^5^the number of included reviews was small and all positive, so publication bias should be considered; ^6^the invalid value (*RR* = 1.0) was included in the confidence interval; ^7^only one study was included, so heterogeneity could not be measured; ^8^most of the included studies were of moderate methodological quality; ^9^incomplete reports and outcome events and selective outcome bias (including adverse reactions, negative results) were not presented or explained. The rating standard of 1 to 9 is referenced in Sects. 4 to 8 in the GRADE guidelines [63–67]

### Data synthesis and meta-analysis

**Fig. 2:**
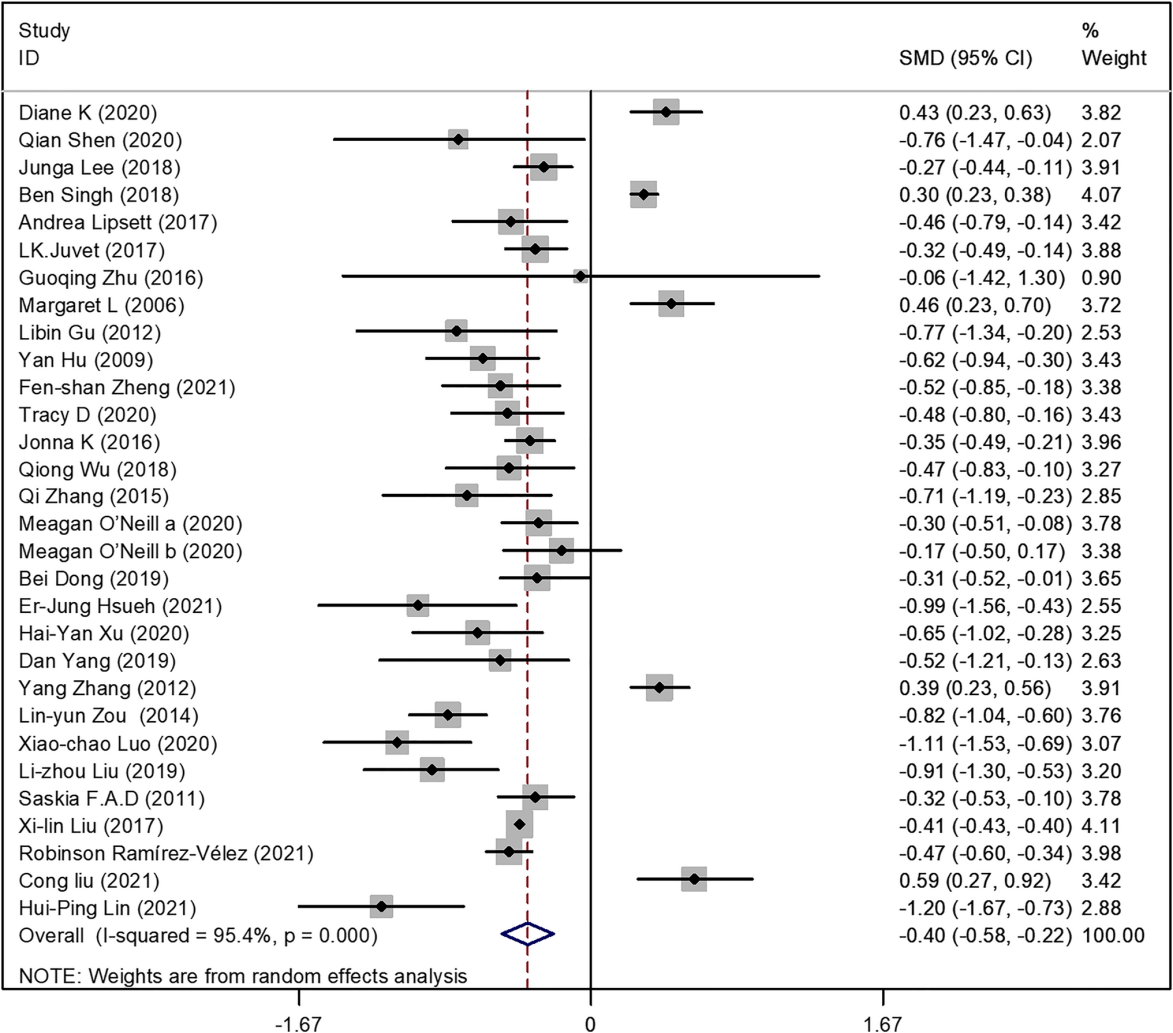
Effect of exercise intervention vs. no exercise or routine methods of care of CRF in breast neoplasms

The overall effects of the exercise interventions in the 29 SRs/meta-analyses indicated that exercise had a moderate effect on the reduction of fatigue in the breast cancer patients in the intervention groups (*SMD* = − 0.40 [95%CI − 0.58, − 0.22]; *P* = 0.0001) (see Fig. 2). However, due to the high heterogeneity among the 29 reviews (*I*^2^ = 95.4%), the overall effect size might be affected by various existing moderating variables. Therefore, the standardized mean differences of the moderating variables were further analyzed to determine the most effective intervention modalities based on the exercise type, frequency, intervention duration, and inclusion/absence of supervision in the intervention protocol. These results are shown in Table 4.

**Table 4:** Quantitative evidence synthesis for fatigue included in the 29 SRs/meta-analyses

Subgroup category	Number of reviews included	Number of original studies included	Number of participants included	Standardized mean difference (95% CI)	*I*-square (%)	Test for overall effect	
						*Z*	*P*
Exercise type							
Aerobic exercise	13	119	12,218	− 0.29 [− 0.56, − 0.02]	16.0	1.98	0.048
Resistance exercise	5	12	1,234	− 0.01 [− 0.31, 0.29]	48.0	0.05	0.958
Yoga	10	92	7,158	− 0.30 [− 0.56, − 0.05]	28.7	2.31	0.021
Tai chi	4	12	904	− 0.19 [− 1.13, 0.76]	75.2	0.38	0.702
Combination exercise	8	67	6,884	− 0.35 [− 0.76, 0.05]	38.8	1.72	0.086
Mind–body exercise	3	11	903	− 0.21 [− 0.63, 0.22]	45.1	0.96	0.336
Other exercise	4	23	2,209	− 0.09 [− 0.45, 0.26]	93.2	0.53	0.599
Overall	47	336	31,510	− 0.23[− 0.35, − 0.11]	95.0	3.84	0.000
Duration of intervention							
Up to two months	4	20	2,067	− 0.55 [− 1.15, 0.06]	34.2	1.76	0.078
Two to six months	5	42	6,877	− 0.23 [− 0.65, 0.19]	59.1	1.06	0.288
More than six months	7	76	3,039	− 0.88 [− 1.59, − 0.17]	42.7	5.57	0.000
Overall	16	128	13,983	− 0.29[− 0.43, − 0.15]	94.8	4.04	0.000
Duration of exercise							
30 to 60 min	5	54	5,028	− 0.81 [− 1.15, − 0.47]	42.3	5.56	0.000
More than 60 min	4	39	3,825	− 0.77 [− 1.04, − 0.50]	0.0	4.66	0.000
Overall	9	93	8,853	− 0.41 [− 0.56, − 0.26]	95.2	5.40	0.000
Frequency of exercise							
Up to three times/week	5	68	4,045	− 0.75 [− 1.58, 0.08]	91.6	1.77	0.076
More than three times/week	4	81	2,672	− 0.65 [− 0.93, − 0.37]	33.0	4.63	0.000
Overall	9	149	6,717	− 0.37[− 0.50, − 0.23]	95.3	5.23	0.000
Supervised							
Yes	7	63	6,511	− 0.48 [− 0.77, − 0.18]	87.0	1.02	0.001
No	6	56	5,376	− 0.21 [− 0.40, − 0.01]	92.2	0.98	0.023
Overall	13	119	11,887	− 0.29 [− 0.43, − 0.14]	95.9	3.90	0.000
Overall included	29	348	33,655	− 0.40 [− 0.58, − 0.22]	95.4	4.87	0.000

#### Exercise type

Seven exercise types were reported in the 29 SRs/meta-analyses, including aerobic exercise, resistance exercise, yoga, mind–body exercise (Pilates and gymnastics), combination exercise (home-based exercise and combined aerobic-resistance exercise), tai chi, and other exercises such as periodic rehabilitation exercise. The subgroup analyses of exercise type showed that both yoga (*SMD* = − 0.30 [95%CI − 0.56, − 0.05]; *I*^2^ = 28.7%; *P* = 0.021) and aerobic exercise (*SMD* = − 0.29 [95%CI − 0.56, − 0.02]; *I*^2^ = 16%; *P* = 0.048) had positive effects on improving CRF in breast cancer patients in the intervention groups compared with those in the control groups receiving routine methods of care. The remaining five exercise types—resistance exercise (*SMD* = − 0.01 [95%CI − 0.31, 0.29]; *I*^2^ = 48%; *P* = 0.958), tai chi (*SMD* = − 0.19 [95%CI − 1.13, 0.76]; *I*^2^ = 75.2%; *P* = 0.702), combination exercise (*SMD* = − 0.35 [95%CI − 0.76, 0.05]; *I*^2^ = 38.8%; *P* = 0.086), mind–body exercise (*SMD* = − 0.21 [95%CI − 0.63, 0.22]; *I*^2^ = 45.1%; *P* = 0.336), and other exercises (*SMD* = − 0.09 [95%CI − 0.45, 0.26]; *I*^2^ = 93.2%; *P* = 0.599)—showed no statistically significant differences between the intervention groups and the control groups using routine methods of care.

#### Duration of the intervention

Intervention duration among the included studies were categorized as: less than 2 months, 2 to 6 months, and more than 6 months. The effect magnitude of the moderating variable of duration had high heterogeneity (*I*^2^ = 94.8%; *P* < 0.000), indicating that the intervention duration could have affected the results of exercise for CRF management in breast cancer patients. The subgroup data indicated that only the intervention duration of more than 6 months had a beneficial effect (*SMD* = − 0.88 [95%CI − 1.59, − 0.17]; *I*^2^ = 42.7%; *P* = 0.000) on CRF compared with routine methods of care. The other intervention duration categories— less than 2 months (*SMD* = − 0.55 [95%CI − 1.15, 0.66]; *I*^2^ = 34.2%; *P* = 0.078) and 2 to 6 months (*SMD* = − 0.23 [95%CI − 0.65, 0.19]; *I*^2^ = 59.1%; *P* = 0.288)—resulted in no statistically significant improvement of CRF.

#### Frequency of exercise

The frequency of exercise included in the 29 SRs/meta-analyses can be categorized as 3 ≤ times per week and > 3 times per week, and the heterogeneity of the combined effect size between the two types was 95.3% (*P* = 0.0001), suggesting that the breast cancer patients’ CRF was affected by the frequency of exercise. The subgroup results of exercise frequency ≤ 3 times per week (*SMD* = − 0.75 [95%CI − 1.58, 0.08]; *I*^2^ = 91.6%; *P* = 0.076) indicated that its effect on CRF in the intervention groups was no statistically different from that in the control groups using routine methods of care, while exercise frequency > 3 times per week (*SMD* = − 0.77 [95%CI − 1.04, − 0.05]; *I*^2^ = 0%; *P* = 0.0001) indicated that the improvement effect was better in the intervention groups compared with the control groups receiving routine methods of care.

#### Duration of exercise

Subgroup analysis revealed that the duration of exercise that lasted 30 to 60 min per session (*SMD* = − 0.81 [95%CI − 1.15, − 0.47]; *I*^2^ = 42.3%; *P* = 0.0001) and 60 min per session (*SMD* = − 0.77 [95%CI − 1.04, − 0.50]; *I*^2^ = 0%; *P* = 0.0001) showed improvement effects on relieving CRF in breast cancer patients in the intervention groups compared with those in the control groups receiving routine methods of care.

#### Inclusion or absence of supervision during exercise

Relevant data on supervised and unsupervised exercise interventions were extracted from the included SRs/meta-analyses and the heterogeneity of effect size was 95.9%, indicating that supervision might impact the effects of exercise on CRF in breast cancer patients. Compared with routine methods of care, exercise interventions relieved the breast cancer patients’ CRF regardless of whether they were supervised while exercising; however, supervised exercise (*SMD* = − 0.48 [95%CI − 0.77, − 0.18]; *I*^2^ = 87%; *P* = 0.001) was shown to produce a larger effect on CRF compared with unsupervised exercise (*SMD* = − 0.21 [95%CI − 0.40, − 0.01]; *I*^2^ = 92.2%; *P* = 0.023).

## Discussion

In this overview, we assessed the methodological and evidence quality of the included SRs/meta-analyses, and additional meta-analyses were performed for the 29 reviews to identify the effects of exercise on CRF in breast cancer patients. The intervention duration, exercise type, duration, and frequency, and whether the exercise intervention was supervised had varying degrees of influence on the effects of exercise on CRF. However, the unsatisfactory methodological quality and level of evidence of the included reviews might affect the reliability of the overview findings on the effects of exercise interventions on CRF in breast cancer survivors, which warrants a prudent interpretation of the study results.

Findings from this study suggested that exercise could be introduced as an effective intervention for CRF management in breast cancer patients. The findings supported the recommendations proposed in some clinical practice guidelines [18, 19, 68], in which exercise was rated and recommended as a beneficial approach to alleviating CRF. However, these guidelines [18, 19, 68] did not mention specific exercise plans. To help further detail the recommendations in the guidelines and facilitate healthcare professionals’ decision-making, subgroup analyses based on the type, frequency, duration, and intensity of exercise were conducted in this study. For exercise type, the study findings suggested that aerobic exercise and yoga were commonly recommended as promising approaches to improving CRF, which is consistent with Lin’s study [69], indicating that yoga can relieve patients’ tension and anxiety and help decrease their fatigue. Yoga is a convenient, easy-to-practice, and safe exercise modality that has been recommended as Grade I evidence by the U.S. National Comprehensive Cancer Network Guidelines [70]. The subgroup analyses results on the duration of exercise indicated that patients who had exercised for more than 6 months achieved the best improvement in fatigue. Our study findings showed that exercising more than three times per week for 30 to 60 min per session was beneficial for CRF alleviation, which is in line with previous research findings [71–73]. The included SRs/meta-analyses also indicated that patients should be encouraged to participate in supervised exercise when conditions permitted, which might lead to a better CRF outcome.

Although quantitative synthesis indicated that exercise interventions can alleviate CRF in breast cancer patients, the findings should be interpreted with caution given the unsatisfactory methodological quality (e.g., lack of reporting a list of exclusion studies and unclear funding resources) and level of evidence identified in the included reviews. For future studies, a list of excluded studies should be provided as an independent appendix to journals to facilitate readers’ understanding of the data selection process and further improve the reliability of the review findings [74]. Moreover, funding sources should be clearly declared in future publications to help readers determine whether funding bias existed. To achieve a comprehensive literature search, future systematic reviews are suggested to identify potential studies by searching not only the commonly used databases but also gray literature retrieval websites to minimize publication bias, for example, Greynet International (http://greynet.org/), the British Library (http://www.b1.uk), and other free grey literature sites such as PLoS. Conference abstracts, book chapters, academic theses and dissertations should also be sources of gray literature. In order to further improve the level of evidence of the included SRs, more original studies with rigorous study designs and detailed descriptions of the intervention protocols (e.g., type, frequency, intensity, and duration of the exercise) are necessary. Nevertheless, there is a need to acknowledge that the results of the unsatisfactory methodological quality of the included SRs may have been related to the selection of the quality appraisal tool. In this overview, nine of the included SRs were published before 2017, while the tool that we used for the quality appraisal (i.e., AMSTAR II) was also updated in 2017, which is an issue that needs to be considered for future overviews.

## Study limitations

This overview has some limitations. Suboptimal methodological quality of some of the includes reviews (e.g., lack of registered protocols, unclear descriptions of data sources) may affect the strength of the evidence. language bias is possible given that only reviews published in Chinese and English were included. Due to the limited number of included reviews, within each current subgroup analysis, further subgroup analyses based on the intervention “dose” (e.g., intervention duration and frequency of each type of exercise intervention) were not conducted, which might, to some extent, limit the generalizability of the review findings to clinical practice.

## Conclusion

Findings from this overview suggested that yoga and aerobic exercise with a long-term practice duration (over 6 months) might benefit CRF alleviation in breast cancer patients. Exercising for at least three times per week for 30 to 60 min per session might be an appropriate dose for alleviating CRF in breast cancer patients. Although existing evidence indicated that exercise interventions have a positive impact on CRF in breast cancer patients, the results should be interpreted with caution due to the limited quantity and unsatisfactory methodological quality and level of evidence of the included SRs/meta-analyses. More rigorously designed large-scale RCTs are needed to provide more robust evidence to specify the exact exercise type, duration, frequency, and intensity to have an optimal effect on CRF in breast cancer patients.

## Supplementary Information

Below is the link to the electronic supplementary material.

Supplementary file1 (DOCX 15 KB)

Supplementary file2 (DOCX 12 KB)

## Data Availability

All authors agreed that the data can be made public and used.
