# Supplementary material for: Effects of exercise interventions on cancer-related fatigue in breast cancer patients: an overview of systematic reviews
Source: Support Care Cancer. 2022 Nov 3;30(12):10421–40. doi: 10.1007/s00520-022-07389-5 (PMC9715478; doi:10.1007/s00520-022-07389-5)
Supplement: Supplementary file 1 — Supplementary file1 (DOCX 15 KB) [file 520_2022_7389_MOESM1_ESM.docx]

**Supplementary file B- List of excluded studies after full text reading and reasons for exclusion**

| Reference | Reason for exclusion |
| --- | --- |
| Ma Z,Guo X,Liu P,et al.Study on the feasibility of exercise as adjunctive therapy for cancer patients.Cancer Prevention and Treatment,2010,37(04):480-482. | Not SRs/reviews |
| Rogers L Q,Hopkins-Price P,Vicari S,et al.A randomized trial to increase physical activity in breast cancer survivors.Medicine&Science in Sports and Exercise,2009,41(4):935. | Not SRs/reviews |
| Matsuoka Y, Tsuji K, Ochi E. Polyunsaturated Fatty Acids, Exercise, and Cancer-Related Fatigue in Breast Cancer Survivors. Front Physiol, 2021,13(12):759280. | Not SRs/reviews |
| Boing L,de Azevedo Guimarães AC,Reis NM,Ribovski M.Atividade física após o diagnóstico do câncer de mama:Revisão sistemática.Motricidade,2016;12(2):155-66. | Not in Chinese or English |
| Wakako O,Ai K,Yukie H,et al.Evaluation of a Support Group for Women with Breast Cancer-Factors Affecting Patients’Satisfaction with Group Experiences and Sense of Supportive Atmosphere.Journal of the Nursing Society of St.Luke,2021,6,19(2):47-53. | Not in Chinese or English |
| Li Q,Gan H,Luo X.Effect of systemic nursing intervention on cancer fatigue in patients with breast cancer chemotherapy.Chinese Journal of Modern Nursing,2015,21(02):129-133. | Exercise intervention was not mentioned |
| de M Alcântara‐Silva TR,Freitas‐Junior R,Freitas NM,Machado GD.Fatigue related to radiotherapy for breast and/or gynaecological cancer:a systematic review.Journal of Clinical Nursing,2013,22(19-20):2679-2686. | Exercise intervention was not mentioned |
| Islam T,Dahlui M,Majid H,et al.Factors associated with return to work of breast cancer survivors:a systematic review.BMC Public Health,2014,14(Suppl 3):S8. | Exercise intervention was not mentioned |
| Minton O,Stone P.How common is fatigue in disease-free breast cancer survivors?A systematic review of the literature.Breast Cancer Research and Treatment,2008,112(1):5-13. | Exercise intervention was not mentioned |
| Zhang J,Xu R,Wang B,et al.Effects of mindfulness-based therapy for patients with breast cancer:A systematic review and meta-analysis.Complementary Therapies in Medicine,2016,26:1-10. | Exercise intervention was not mentioned |
| Barbagelata K,Eadi J,Mcnamara M,et al.Aquatic Therapy Reduces Pain and Fatigue in Breast Cancer Survivors:A Systematic Review.Rehabilitation Oncology,2021,publish ahead of print. | Exercise intervention was not mentioned |
| Furmaniak AC,Menig M,Markes MH.Exercise for women receiving adjuvant therapy for breast cancer.Cochrane Database Syst Rev,2016,21;9(9):CD005001. | No RCTs included |
