# Supplementary material for: Effects of exercise interventions on cancer-related fatigue in breast cancer patients: an overview of systematic reviews
Source: Support Care Cancer. 2022 Nov 3;30(12):10421–40. doi: 10.1007/s00520-022-07389-5 (PMC9715478; doi:10.1007/s00520-022-07389-5)
Supplement: Supplementary file 2 — Supplementary file2 (DOCX 12 KB) [file 520_2022_7389_MOESM2_ESM.docx]

**Supplementary file A – Selected search algorithms**

**1.PubMed**

((((((((((((((systematic review[Title/Abstract]) OR (systematic appraisal [Title/Abstract])) OR (systematic assessment[Title/Abstract])) OR (SR[Title/Abstract])) OR (systematic evaluation[Title/Abstract])) OR (meta-analysis[Title/Abstract])) OR (meta[Title/Abstract])) OR (meta-analyses[Title/Abstract])) OR (meta analysis[Title/Abstract])) OR (review[Title/Abstract])) OR (systematic overview[Title/Abstract])) OR (systematic review and meta-analysis[Title/Abstract])) AND (((fatigue[Title/Abstract]) OR (cancer related fatigue[Title/Abstract])) OR (CRF[Title/Abstract])))AND (((((exercise[Title/Abstract]) OR (physical[Title/Abstract])) OR (activity[Title/Abstract])) OR (exercise traning[Title/Abstract])) OR (aerobic exercise[Title/Abstract])))AND (((((breast cancer[Title/Abstract]) OR (breast neoplasms[Title/Abstract])) OR (breast tumor[Title/Abstract])) OR (mammary cancer[Title/Abstract])) OR (breast carcinoma[Title/Abstract]))

**2.Embase**

(‘systematic review’:ti,ab,kw OR ‘systematic appraisal’:ti,ab,kw OR ‘systematic assessment’:ti,ab,kw OR ‘systematic evaluation’:ti,ab,kw OR meta:ti,ab,kw OR ‘meta analyses’:ti,ab,kw OR ‘meta analysis’:ti,ab,kw OR ‘review literature’:ti,ab,kw OR ‘systematic overview’:ti,ab,kw OR ‘systematic review and meta-analysis’:ti,ab,kw)AND (fatigue:ti,ab,kw OR ‘cancer related fatigue’:ti,ab,kw OR crf:ti,ab,kw) AND(exercise:ti,ab,kw OR physical:ti,ab,kw OR activity:ti,ab,kw OR ‘exercise traning’:ti,ab,kw OR ‘aerobic exercise’:ti,ab,kw) AND (‘breast cancer’:ti,ab,kw OR ‘breast neoplasms’:ti,ab,kw OR ‘breast tumor’:ti,ab,kw OR ‘mammary cancer’:ti,ab,kw OR ‘breast carcinoma’:ti,ab,kw)
